# The Role of Perceived Loneliness in Youth Addictive Behaviors: Cross-National Survey Study

**DOI:** 10.2196/14035

**Published:** 2020-01-02

**Authors:** Iina Savolainen, Atte Oksanen, Markus Kaakinen, Anu Sirola, Hye-Jin Paek

**Affiliations:** 1 Faculty of Social Sciences, Tampere University Tampere Finland; 2 Institute of Criminology and Legal Policy, University of Helsinki Helsinki Finland; 3 Hanyang University Ansan Republic of Korea

**Keywords:** youth, problem behavior, excessive alcohol consumption, internet, gambling, loneliness

## Abstract

**Background:**

In the ever-growing and technologically advancing world, an increasing amount of social interaction takes place through the Web. With this change, loneliness is becoming an unprecedented societal issue, making youth more susceptible to various physical and mental health problems. This societal change also influences the dynamics of addiction.

**Objective:**

Employing the cognitive discrepancy loneliness model, this study aimed to provide a social psychological perspective on youth addictions.

**Methods:**

A comprehensive survey was used to collect data from American (N=1212; mean 20.05, SD 3.19; 608/1212, 50.17% women), South Korean (N=1192; mean 20.61, SD 3.24; 601/1192, 50.42% women), and Finnish (N=1200; mean 21.29, SD 2.85; 600/1200, 50.00% women) youths aged 15 to 25 years. Perceived loneliness was assessed with the 3-item Loneliness Scale. A total of 3 addictive behaviors were measured, including excessive alcohol use, compulsive internet use, and problem gambling. A total of 2 separate models using linear regression analyses were estimated for each country to examine the association between perceived loneliness and addiction.

**Results:**

Loneliness was significantly related to only compulsive internet use among the youth in all 3 countries (*P*<.001 in the United States, South Korea, and Finland). In the South Korean sample, the association remained significant with excessive alcohol use (*P*<.001) and problem gambling (*P*<.001), even after controlling for potentially confounding psychological variables.

**Conclusions:**

The findings reveal existing differences between youths who spend excessive amounts of time online and those who engage in other types of addictive behaviors. Experiencing loneliness is consistently linked to compulsive internet use across countries, although different underlying factors may explain other forms of addiction. These findings provide a deeper understanding in the mechanisms of youth addiction and can help improve prevention and intervention work, especially in terms of compulsive internet use.

## Introduction

### Background

Addictive behaviors are a continuous burden to public health, affecting millions of individuals globally. Adolescents and emerging adults are particularly vulnerable to the harms of addictions, as they can disrupt healthy development [1], cause long-term health defects [2], damage social relationships [3], and devastate future financial competences [4]. It is widely known that youths typically engage in behaviors that are harmful, sometimes deviant, and place them at risk for injury, disease, and even unintentional mortality [5,6]. Hence, youths can be considered more susceptible to initiating at least some type of addictive behavior during the periods of adolescence and emerging adulthood.

Addictive behaviors are difficult to prevent and address because they originate progressively, are often concealed by the individual affected, and impact the person comprehensively on biological, psychological, and social levels [7]. Commonly, addiction is associated with substance misuse, and alcohol is still the most frequently used and misused substance among the youth [8]. Studies have consistently shown that adolescents are most likely to engage in heavy episodic alcohol consumption [9,10]. An additional challenge of addiction is that, in addition to substances, a wide range of objects and activities exist to which one can become addicted [11]. The 5th edition of the Diagnostic and Statistical Manual of Mental Disorders now recognizes behavioral addictions, such as excessive gambling, under nonsubstance-related disorders [12].

Gambling has increased its popularity as a recreational activity among the youth during the past years [13]. Even though many laws and regulations prohibit underage individuals from engaging in gambling activities, new gambling technologies, especially online gambling, allow easy and constant access to the activities, often without viable age restrictions [4,14]. Past research has found that gambling activities are typically initiated at the age of 16 years, whereas gambling problems are most common among individuals between ages 18 and 24 years [15]. Moreover, a recent study discovered that 12.3% of the youth across 5 continents qualify as problem gamblers [16].

Internet use is another everyday behavior that can become excessive or compulsive and cause significant harm to an individual over time [17]. The internet has become practically an inseparable part of people’s lives, and although its benefits are preeminent, it can interfere normal functioning and routines when used excessively. Past research on excessive internet use has identified associations with negative adolescent psychosocial development [18], mood abnormalities [19], and lower school performance [20]. Adolescents are more likely to engage in compulsive internet use, become addicted to the use, and are found to be more vulnerable to its negative effects [21].

As technology keeps advancing rapidly, it simultaneously changes the structure of the social world. People must adapt to these societal progresses quickly, and oftentimes, youths are leading this adaptation process. As modern devices allow individuals to stay connected continuously, find contacts across unprecedented distances, and even create virtual companionships, they are paradoxically becoming more disconnected from each other. This manifests in lessened face-to-face interaction with physical social connections [22] and allows for new types of social exclusion such as cyber bullying [23]. This societal change might further result in increased perceived loneliness, specifically among young individuals, to whom peer relationships are salient and exploring different social identities is relevant [24,25]. Loneliness experienced in youth can have significant consequences in terms of mental health and overall well-being [26], and a potential reason for youth addictive behaviors may lie in perceived loneliness.

Loneliness is a common and distressing experience that people typically attempt to avoid [27]. Loneliness has been challenging to define, and different typologies of it have been distinguished. Past research has generally described loneliness as a subjective feeling of an individual that occurs as a natural response to certain situations [28]. It is often accompanied by feelings of anxiety, emptiness, and social isolation [28,29]. Many background variables are associated with loneliness, but the subjective evaluation of one’s realized social relationships has been shown to be one of the most important determinants of experiencing loneliness [30].

The cognitive discrepancy model by Perlman and Peplau [27] offers a blueprint for social psychological theory of loneliness, and it is broadly used in conceptualizing loneliness. The model states that individuals experience loneliness when their personal network of social relations is either quantitatively or qualitatively deficient. When an individual’s expectations of his or her interpersonal interactions do not meet with the existing ones, it results in loneliness [27].

Loneliness can have severe and long-lasting consequences. Past research has identified loneliness as a risk factor in a range of destructive health behaviors. For instance, social isolation and loneliness are associated with a greater risk of smoking and being inactive [31]. Previous research has also linked loneliness to problematic internet use [19]. To a more severe effect, loneliness and social isolation have been consistently found to result in higher likelihood of premature mortality, emphasizing social relationships’ fundamental role in people’s life [26]. Although much research on the effects of loneliness examines adult populations, similar effects also exist among young individuals: A study on college freshmen concluded that loneliness was associated with a lower immune response, poorer sleep quality, and elevations in cortisol levels [32]. Another study found indications that loneliness increases the risk of substance use among adolescents in the United States and Russia [33].

In this study, we examine the association of loneliness with a range of youth addictive behaviors in 3 diverse countries: the United States, South Korea, and Finland. These countries were chosen for their distinct cultural features [34] while being technologically analogous, advanced, and affluent. However, rich environmental and social resources do not safeguard youths from experiencing negative emotions and engaging in destructive behaviors: youths from these cultures experience declines in global life-satisfaction and well-being in the onset and progression of adolescence [35]. In addition, negative social experiences (eg, loneliness, discrimination, and self-concept) are related to lower subjective well-being among the youth in these countries [35].

Comparing the 3 countries in terms of addictive behaviors is also feasible: Recent statistics indicate that up to 95% of the youths in these countries are active internet users [36]. Alcohol consumption among the youth in the United States and Finland is comparably high, whereas, in comparison, South Korean youths consume less alcohol [37]. Similarly, the United States and Finland show average prevalence of problem gambling, whereas the rate is below average in South Korea [38].

### Objectives

Guided by the social psychological cognitive discrepancy loneliness model [27] as our theoretical framework, and existing literature on loneliness and well-being [19,26,31], this study aimed to explore how influential perceived loneliness is in youth addictive behaviors. More specifically, we investigated if loneliness is related to a range of addictions—excessive alcohol use, compulsive internet use, and problem gambling in varying ways—and if the examined relationships are consistent across different cultures.

## Methods

### Data Collection Procedure

Data were collected using an online survey from March to April 2017 in Finland, in January 2018 in the United States, and in February 2018 in South Korea. The study was originally conducted in Finland and expanded to the United States and South Korea for cross-national comparison. All datasets were collected with LimeSurvey software (LimeSurvey GmbH) using identical survey formats. The surveys were optimized for both computers and mobile devices. The original survey was in Finnish. It was translated into English by bilingual professional-level translators and back-translated again to ensure consistency and accurate matching of all items. The English survey was translated to Korean, which was also back-translated to English by bilingual, professional-level translators fluent in both English and Korean. The survey included measures for all target variables, including excessive alcohol use, problem gambling, compulsive internet use, and perceived loneliness. Existing translations of the validated target measures were used when available. To fit the cultural settings as accurately as possible in all 3 countries, the formatting and terminology used were slightly modified for some items [39]. The average survey response time was similar across the 3 countries, with each version of the survey taking about 15 min to complete.

### Participants

The samples consisted of a total of 1212 American (mean 20.05, SD 3.19; 608/1212, 50.17% women), 1192 South Korean (mean 20.61, SD 3.24; 601/1192, 50.42% women), and 1200 Finnish (mean 21.29, SD 2.85; 600/1200, 50.00% women) participants aged 15 to 25 years. All samples were demographically balanced in terms of age, gender, and living area. The participants were recruited from a pool of volunteers administered by using Dynata: a global data collection provider that manages online panels in several countries. By managing quotas via balanced start methodology, it is possible to attain data that are consistent and match the demographic profile of each examined country. Owing to the convenience sampling with set quotas, nonresponse rates cannot be reported. There are no missing data. The local Academic Ethics Committee approved this research before implementation. Participation in the study was anonymous and voluntary. All ethical guidelines were followed.

### Measures

The participants were asked to provide some key demographic information, including age, gender, and living situation. This information was acquired through individual questions in the surveys. Living situation was asked with the inquiry: Are you currently... with answer choices ranging from “Living alone?” to “Living with parents” or “Other.” For the analysis, living situation was recoded into a “Living alone” dummy variable (0=no, 1=yes). This variable was included in the analyses to account for the participants’ social disposition through living situation.

To analyze the relationship between loneliness and a range of addictive behaviors, we employed validated and reliable measures for all target addictive behaviors. To identify excessive alcohol use among the youth, the 3-item Alcohol Use Disorders Identification Test-Consumption (AUDIT-C) was used. The AUDIT-C is a brief, reliable, and effective way of measuring hazardous drinking habits in a survey [40]. The questionnaire is validated for several populations and has been found to perform well in the screening of alcohol misuse across studies [41]. The South Oaks Gambling Screen (SOGS) was utilized to measure the frequency and intensity of gambling behavior. The SOGS is a reliable (alpha=.90 in the US sample, alpha=.82 in the Korean sample, and alpha=.89 in the Finnish sample) and regularly used measure to screen for pathological gambling behavior [42]. Some of the test items were slightly modified to accommodate for cultural variations in gambling practices.

The Compulsive Internet Use Scale (CIUS) was used to measure compulsive internet use. The scale measures internet use behavior effectively and evaluates whether usage is compulsive [43]. The scale has good psychometric properties and reliability statistics (alpha=.95 in the United States, alpha=.95 in South Korea, and alpha=.93 in Finland). The CIUS consists of 14 items, each of which targets the consequences and states of mind involved in internet use. Responses range on a 5-point scale from 0 (never) to 4 (very often), with a higher score indicating compulsive internet use. The 3-item Loneliness Scale was used to measure perceived loneliness. The scale is a short measure originally adapted from the standard Loneliness Scale, the Revised UCLA Loneliness Scale [44]. It was designed for efficiently measuring loneliness in large-scale social surveys. We applied the short measure to keep the survey brief for young respondents. The short measure has been shown to be internally consistent with both discriminant and concurrent validity, and its results are comparable with those of the full measure [45]. The 3-item scale asks, “How often do you feel” (1) “that you lack companionship?” (2) “left out?” (3) “isolated from others?” Answer choices provided are: “1=hardly ever; 2=some of the time; and 3=often.” The variable was turned into a sum composite.

Possible confounding variables were taken into consideration*.* We identified 3 variables that were additionally included in the analyses because they may be confounded with loneliness; psychological distress, belonging to a friendship group, and belonging to an online community [46,47]. In addition, belonging to a friendship group and perceived loneliness were inversely correlated, making belonging to a friendship group a feasible control variable. Psychological distress was measured with the General Health Questionnaire-12. The scale has good internal consistency and reliability (alpha=.88 in all 3 country samples). Belonginess to a friendship group and an online community were evaluated with the item asking: How strongly do you feel you belong to the following? Designated groups and communities were indicated as “A friendship group? and “An online community?” Answers were provided with a 10-point Likert scale (1=not at all, 10=very strongly).

### Statistical Analysis

Analyses were conducted by first calculating descriptive statistics for all variables. These are reported as means and standard deviations for continuous variables and as frequencies (n) and relational proportions (%) for categorical variables. We conducted Kruskal-Wallis tests to compare whether statistically significant mean differences exist between the 3 countries in terms of perceived loneliness, excessive alcohol use, compulsive internet use, and problem gambling.

A total of 2 separate multiple linear regression models were estimated for each country. The assumptions of ordinary least squares (OLS) regression were met, but because of heteroscedasticity of the residuals, we carried out the analyses by producing robust SEs [48,49]. The first model included 3 regression estimates for each addictive behavior in each country, with predictors and demographic factors. The second model included 3 regression estimates for each addictive behavior in each country, with predictors and demographic factors, controlling for the 3 potentially confounding psychological factors; belonging to a friendship group, belonging to an online community, and psychological distress. We also tested for potentially confounding interactions, including age and gender, but these did not change the main results concerning the relationship between loneliness and addictive behaviors. They were, hence, left out.

Tests of collinearity were run after each model to assess the variance inflation rate. No collinearity was observed in the models, with mean variance inflation factors ranging from 1.22 to 1.26, indicating only moderate correlation [50,51]. Correlations between dependent variables were also investigated. These are reported in Multimedia Appendix 1. To confirm the consistency of findings, the analyses were repeated (1) after removing potential outliers from the dataset, (2) with zero-inflated Poisson regression, and (3) with logistic regression analysis by using recommended cutoff scores for each addiction measure: ≥3 for the AUDIT-C [40,52], ≥21 for the CIUS [53,54], and ≥8 for the SOGS [55,56]. Zero-inflated Poisson regression has been noted as the most reliable regression model and especially suitable for addiction research having excess zeros in the data. This also solves problems related to cutoff scores that have been discussed in addiction research [57].

## Results

### Descriptive Statistics

The descriptive statistics (presented in detail in Tables 1 and 2) show that perceived loneliness was proportionately high in the United States and Finland (mean 5.52 in both countries), followed by South Korea (mean 5.23). These mean differences were statistically significant when comparing the United States and Finland with South Korea (*P*<.001). Excessive alcohol use was highest in Finland (mean 4.14), followed by South Korea (mean 3.85) and the United States (mean 2.29). These mean differences were significant between the United States and South Korea (*P*<.001), the United States and Finland (*P*<.001), and between Finland and South Korea (*P*<.001). Compulsive internet use was highest among youths in South Korea (mean 23.13), followed by American (mean 21.72) and Finnish youths (mean 18.80). These mean differences were significant between the United States and South Korea (*P*<.001) and between South Korea and Finland (*P*<.001). Mean problem gambling scores were highest in Finland (mean 1.60), followed by the United States (mean 1.34) and South Korea (mean 1.04). These mean differences were significant between all 3 countries (*P*<.001 in each country comparison).

**Table 1:** Descriptive statistics.

Variable	United States		South Korea		Finland	
	Mean (SD)	Range	Mean (SD)	Range	Mean (SD)	Range
Excessive alcohol use	2.29 (2.69)	0-13	3.85 (3.51)	0-13	4.14 (2.98)	0-13
Compulsive internet use	21.72 (13.54)	0-56	23.13 (12.81)	0-–56	18.80 (11.13)	0-56
Problem gambling	1.34 (2.69)	0-20	1.04 (2.86)	0-20	1.60 (2.56)	0-20
Perceived loneliness	5.52 (1.86)	3-9	5.23 (1.73)	3-9	5.52 (1.78)	3-9
Belonging to friends	6.72 (2.62)	1-10	6.80 (2.21)	1-10	6.83 (2.48)	1-10
Belonging online^a^	5.38 (2.69)	1-10	4.38 (2.48)	1-10	5.03 (2.60)	1-10
Psychological distress	13.49 (6.75)	0-36	13.61 (5.98)	0-36	14.15 (6.34)	0-36
Age	20.05 (3.19)	15-25	20.61 (3.24)	15-25	21.29 (2.85)	15-25

^a^Belonging to an online community.

**Table 2:** Descriptive statistics of categorical variables.

Variables		United States, n (%)	South Korea, n (%)	Finland, n (%)
**Gender**				
	Male	604 (49.83)	591 (49.58)	600 (50.0)
	Female	608 (50.17)	601 (50.42)	600 (50.0)
Living alone (yes)		136 (11.2)	139 (11.7)	396 (33.0)

### Results of Model 1

According to our model 1, loneliness was significantly associated with alcohol use in the United States (beta=.12; *P*<.001) and South Korea (beta=.06; *P*=.02), but not in Finland (beta=.01; *P*=.82). Out of the demographic factors, higher age was significantly related to excessive alcohol use across the 3 countries. The male gender predicted excessive alcohol use in the United States (beta=–.10; *P*<.001) and Finland (beta=–.06; *P*=.03) but not in South Korea (beta=–.04; *P*=.13). Living alone was a statistically significant predictor of excessive alcohol use in South Korea (beta=.10; *P*=.001; see Table 3). Compulsive internet use was significantly associated with loneliness in all countries (beta=.41, *P*<.001 in the United States; beta=.30, *P*<.001 in South Korea; and beta=.28, *P*<.001 in Finland). Lower age was a significant predictor of compulsive internet use in South Korea (beta=–.16; *P*<.001) and Finland (beta=–.13; *P*<.001). In the United States, the male gender (beta=–.05; *P*=.04) and living alone (beta=.06; *P*=.03) predicted compulsive internet use (Table 4).

**Table 3:** Main effects of ordinary least squares regression models with robust standard errors predicting excessive alcohol use (Alcohol Use Disorders Identification Test-Consumption) in 3 countries.

Model		United States				South Korea				Finland			
		B^a^	SE	Beta	*P* value	B	SE	Beta	*P* value	B	SE	Beta	*P* value
**Model 1**													
	Constant	–4.9^b^	0.50^b^	—^c^	<.001^b^	–5.1^b^	0.69^b^	—	<.001^b^	–.51	0.67	—	.44
	Perceived loneliness	.18^b^	0.04^b^	.12^b^	<.001^b^	.13^b^	0.06^b^	.06^b^	.02^b^	0.01	0.05	0.01	.82
	Age (years)	.35^b^	0.02^b^	.41^b^	<.001^b^	.42^b^	0.03^b^	.38^b^	<.001^b^	.24^b^	0.03^b^	.23^b^	<.001^b^
	Gender^d^	–.53^b^	0.14^b^	–.10^b^	<.001^b^	–.28	0.18	–.04	.13	–.37^b^	0.17^b^	–.06^b^	.034^b^
	Living alone^e^	0.27	0.24	0.03	.27	1.1^b^	0.31^b^	.10^b^	.001^b^	0.28	0.19	0.04	.15
	R^2^	0.21	—	—	—	0.18	—	—	—	0.06	—	—	—
**Model 2**													
	Constant	–5.5	0.58	—	>.001	–7.1^b^	0.90^b^	—	<.001^b^	–1.9	0.83	—	.02
	Perceived loneliness	0.07	0.04	0.05	.13	.22^b^	0.06^b^	.11^b^	.001^b^	–.02	0.06	–.01	.68
	Age (years)	.33^b^	0.02^b^	.40^b^	<.001^b^	.42^b^	0.03^b^	.39^b^	<.001^b^	.22^b^	0.03^b^	.21^b^	<.001^b^
	Gender^d^	–.56^b^	0.14^b^	–.10^b^	<.001^b^	–.33	0.19	–.05	.07	–.70^b^	0.17^b^	–.12^b^	<.001^b^
	Living alone^e^	0.26	0.24	0.03	.27	.95^b^	0.31^b^	.09^b^	.003^b^	0.3	0.18	0.05	.11
	Belonging to friends	0.06	0.03	0.06	.06	.25^b^	0.05^b^	.16^b^	<.001^b^	.24^b^	0.04^b^	.20^b^	<.001^b^
	Belonging to an online community	0.02	0.03	0.02	.52	–.08	0.04	–.06	.05	–.12^b^	0.03^b^	–.10^b^	.001^b^
	Psychological distress	.08^b^	0.01^b^	.20^b^	<.001^b^	0.01	0.02	0.02	.59	.10^b^	0.01^b^	.21^b^	<.001^b^
	R^2^	0.24	—	—	—	0.2	—	—	—	0.12	—	—	—

^a^Unstandardized regression coefficient.

^b^Statistically significant results (*P*<.05).

^c^Value not applicable.

^d^Gender reference category male.

^e^Living alone measured with a dummy variable (0=No, 1=Yes).

**Table 4:** Main effects of ordinary least squares regression models with robust standard errors predicting compulsive internet use (Compulsive Internet Use Scale) in 3 countries.

Model		United Sates				South Korea				Finland			
		B^a^	SE	Beta	*P* value	B	SE	Beta	*P* value	B	SE	Beta	*P* value
**Model 1**													
	Constant	8.2^b^	2.6^b^	—^c^	.002^b^	23.6^b^	2.5^b^	—	<.001^b^	19.8^b^	2.6^b^	—	<.001^b^
	Perceived loneliness	3.02^b^	0.21^b^	.41^b^	<.001^b^	2.2^b^	0.22^b^	.30^b^	<.001^b^	1.8^b^	0.18^b^	.28^b^	<.001^b^
	Age (years)	–.06	0.11	–.01	.58	–.63^b^	0.11^b^	–.16^b^	<.001^b^	–.51^b^	0.11^b^	–.13^b^	<.001^b^
	Gender^d^	–1.5^b^	0.72^b^	–.05^b^	.04^b^	0.54	0.7	0.02	.44	0.24	0.62	0.01	.7
	Living alone^e^	2.7^b^	1.2^b^	.06^b^	.03^b^	0.78	1.1	0.02	.48	–.40	0.68	–.02	.56
	R^2^	0.18	—	—	—	0.11	—	—	—	0.09	—	—	—
**Model 2**													
	Constant	–3.0	2.9	—	.3	6.3	3	—	.04	9.3	3.1	—	.003
	Perceived loneliness	2.2^b^	0.23^b^	.30^b^	<.001^b^	1.6^b^	0.24^b^	.21^b^	<.001^b^	1.4^b^	0.21^b^	.23^b^	<.001^b^
	Age (years)	–.07	0.11	–.02	.52	–.35^b^	0.11^b^	–.09^b^	.001^b^	–.34^b^	0.11^b^	–.09^b^	.002^b^
	Gender^d^	–1.2	0.68	–.04	.09	0.26	0.67	0.01	.7	0.64	0.63	0.03	.31
	Living alone^e^	2.5^b^	1.1^b^	.06^b^	.02^b^	0.62	1	0.02	.55	–.52	0.66	–.02	.43
	Belonging to friends	0.13	0.15	0.02	.41	.51^b^	0.18^b^	.09^b^	.01^b^	–.12	0.14	–.03	.37
	Belonging to an online community	1.4^b^	0.14^b^	.27^b^	<.001^b^	1.2^b^	0.16^b^	.24^b^	<.001^b^	.98^b^	0.13^b^	.23^b^	<.001^b^
	Psychological distress	.54^b^	0.06^b^	.27^b^	<.001^b^	.48^b^	0.08^b^	.22^b^	<.001^b^	.29^b^	0.06^b^	.16^b^	<.001^b^
	R^2^	0.3	—	—	—	0.19	—	—	—	0.16	—	—	—

^a^Unstandardized regression coefficient.

^b^Statistically significant results (*P*<.05).

^c^Value not applicable.

^d^Gender reference category male.

^e^Living alone measured with a dummy variable (0=No, 1=Yes).

In addition, in model 1, problem gambling was significantly related to loneliness in all 3 countries: in the United States (beta=.10; *P*=.001), South Korea (beta=.18; *P*<.001), and Finland (beta=.11; *P*=.001). Age was significantly related to problem gambling in the United States and South Korea, but this association was inverse. In the United States, higher age associated with problem gambling (beta=.15; *P*<.001), whereas in South Korea, lower age was associated with problem gambling (beta=–.09; *P*=.01). Across the 3 countries, the male gender was highly related to problem gambling (beta=–.14; *P*<.001 in the United States; beta=–.16; *P*<.001 in South Korea; and beta=–.26; *P*<.001 in Finland). In the United States, living alone was a significant predictor of problem gambling (beta=.13; *P*=.001; Table 5).

**Table 5:** Main effects of ordinary least squares regression models with robust standard errors predicting problem gambling (South Oaks Gambling Screen) in 3 countries.

Model		United States				South Korea				Finland			
		B^a^	SE	Beta	*P* value	B	SE	Beta	*P* value	B	SE	Beta	*P* value
**Model 1**													
	Constant	–.91	0.54	—^b^	.1	1.7^c^	0.40^c^	—	<.001^c^	2.4^c^	0.60^c^	—	<.001^c^
	Perceived loneliness	.14^c^	0.04^c^	.10^c^	.001^c^	.21^c^	0.04^c^	.18^c^	<.001^c^	.16^c^	0.04^c^	.11^c^	.001^c^
	Age (years)	.13^c^	0.03^c^	.15^c^	<.001^c^	–.05^c^	0.02^c^	–.09^c^	.01^c^	0.01	0.03	0.01	.67
	Gender^d^	–.76^c^	0.15^c^	–.14^c^	<.001^c^	–.63^c^	0.11^c^	–.16^c^	<.001^c^	–1.3^c^	0.15^c^	–.26^c^	<.001^c^
	Living alone^e^	1.1^c^	0.32^c^	.13^c^	.001^c^	0.42	0.25	0.07	.09	0.05	0.17	0.01	.76
	R^2^	0.08	—	—	—	0.06	—	—	—	0.07	—	—	—
**Model 2**													
	Constant	–1.7^c^	0.77^c^	—	.02^c^	0.89	0.64	—	.17	3.1^c^	1.0^c^	—	.002^c^
	Perceived loneliness	0.01	0.05	0	.91	.14^c^	0.04^c^	.12^c^	.001^c^	0.01	0.06	0.01	.81
	Age (years)	.12^c^	0.02^c^	.14^c^	<.001^c^	–.03	0.02	–.05	.1	0	0.03	0	.94
	Gender^d^	–.74^c^	0.14^c^	–.14^c^	<.001^c^	–.63^c^	0.12^c^	–.16^c^	<.001^c^	–1.4^c^	0.16^c^	–.28^c^	<.001^c^
	Living alone^e^	1.0^c^	0.32^c^	.12^c^	.001^c^	0.45	0.24	0.07	.07	0.06	0.17	0.01	.75
	Belong to friends	–.02	0.03	–.02	.47	–.03	0.03	–.03	.35	–.07	0.04	–.07	.11
	Belonging to an online community	.16^c^	0.03^c^	.16^c^	<.001^c^	.12^c^	0.03^c^	.14^c^	<.001^c^	0.01	0.03	0.01	.83
	Psychological distress	.08^c^	0.01^c^	.20^c^	<.001^c^	.03^c^	0.01^c^	.10^c^	.02^c^	.07^c^	0.02^c^	.17^c^	<.001^c^
	Adjusted R^2^	0.13	—	—	—	0.08	—	—	—	0.09	—	—	—

^a^Unstandardized regression coefficient.

^b^Value not applicable.

^c^Statistically significant results (*P*<.05).

^d^Gender reference category male.

^e^Living alone measured with a dummy variable (0=No, 1=Yes).

### Results of Model 2

Model 2 added the potentially confounding factors to the analyses. According to the model, loneliness was significantly associated with alcohol use only within the South Korean sample (beta=.11; *P*=.001). Higher age remained a significant predictor of excessive alcohol use in all 3 countries (beta=.40; *P*<.001 in the United States; beta=.39; *P*<.001 in South Korea; and beta=.21; *P*<.001 in Finland). Similarly, the male gender continued to predict excessive alcohol use in the United States (beta=–.10, *P*<.001) and Finland (beta=–.12; *P*<.001). Living alone endured the confounds and remained a statistically significant predictor of excessive alcohol use in South Korea (beta=.09; *P*=.003).

The relationship between loneliness and compulsive internet use remained highly significant in all 3 countries: in the United States (beta=.30; *P*<.001), South Korea (beta=.21; *P*<.001), and Finland (beta=.23; *P*<.001). Lower age was also a significant predictor of compulsive internet use in South Korea (beta=–.09; *P*=.001) and Finland (beta=–.09; *P*=.002). The effects of gender were no longer observed (beta=–.04, *P*=.09 in the United States; beta=.01, *P*=.70 in South Korea; and beta=.03, *P*=.31 in Finland). Living alone persisted as a predictor of compulsive internet use among youths in the United States (beta=.06; *P*=.02).

With the added confounds, loneliness continued to significantly associate with problem gambling only within the South Korean sample (beta=.12; *P*=.001). In the United States, higher age remained associated with problem gambling (beta=.14; *P*<.001), whereas in South Korea, the effect of age was no longer significant (beta=–.05; *P*=.10). Furthermore, in model 2, the male gender was strongly related to problem gambling in all 3 countries (beta=–.14, *P*<.001 in the United States; beta=–.16, *P*<.001 in South Korea; and beta=–.28, *P*<.001 in Finland). In addition, living alone remained a significant predictor of problem gambling within youths in the United States (beta=.12; *P*=.001). Full models are reported in Tables 3 to 5.

The additional OLS regression analyses with removed potential outliers reproduced the above findings. Only excessive alcohol use became significant in model 2 of the US sample (beta=.07; *P*=.02). The supplementary robustness checks of the analyses were done by running the models with zero-inflated Poisson regression (Multimedia Appendix 1) and logistic regression analysis using cutoff scores for addictive behaviors (Multimedia Appendix 2). These analyses showed that loneliness was associated with compulsive internet use in all countries, whereas excessive alcohol use and problem gambling remained significant only in South Korea.

## Discussion

### Principal Findings

Analyzing data from 3 different countries, this study examined the association of perceived loneliness with different addictive behaviors among adolescents and young adults. Loneliness was consistently associated with compulsive internet use across the 3 countries: the United States, South Korea, and Finland. An association between loneliness and excessive alcohol use was observed among youths in South Korea. Similarly, loneliness was related to problem gambling among South Korean youths. These findings were consistent when testing for confounding interactions of age and gender, indicating that to an extent, loneliness is associated with addiction, irrespective of gender or age. These results provide support for previous research [19,21] and further emphasize that loneliness is a potential risk factor particularly in compulsive internet use. Youths who experience loneliness seem more likely to follow online-oriented addiction trajectories across countries. Those youths who are more fulfilled with their interpersonal relationships might experiment with other types of disruptive behaviors, such as problem gambling and excessive alcohol consumption.

Following the cognitive discrepancy loneliness model, we suggest that lonely youths might feel as though their social needs are not adequately satisfied, which manifests in spending excessive amounts of time online. It is also possible that these youths would search for meaningful, like-minded social interactions [58,59] and identities through different online communities [60].

### Cultural Significance of Findings

The extent to which youths engage in different types of addictive behaviors might reveal explanatory differences about youths in their cultural settings. Within this study, we found similarities between youths in terms of loneliness and engagement in internet use. Prevalence statistics indicate that the youth in these 3 countries are skilled internet users [36], and this finding further suggests that the internet is particularly attractive to lonely youth. However, we propose that the youth living in diverse settings have different motivations for spending excessive time online: for the individualistic American and Finnish youths, the internet is a place of expressing oneself and building a presence that is loosely connected to other users, whereas collectivistic South Korean youths might seek for virtual companionship with whom to share mutual values and support with [34]. In addition, the results indicate that compulsive internet use is highly associated with younger age, emphasizing the importance of early interventions.

In South Korea, loneliness was also found to be significantly related to excessive alcohol use and problem gambling. This result might indicate that perceived loneliness among collectivistic South Korean youth results in more serious consequences than among youths in the United States and Finland [61]. Addictive behaviors may further function as a way of coping with uncertainty caused by the deviation away from the South Korean societal status quo. These results may also reflect current behavioral trends prevailing among youths across the world. Extensive Health Behaviour in School-Aged Children data found that adolescents in the United States and Finland have become more uniform in their drunkenness frequency, with overall alcohol consumption among adolescent age groups decreasing slightly [62], whereas a nationwide research on South Korean youths’ health behavior recognized the increasing rate of alcohol use among the youth as a major issue [61].

In the United States and Finland, the social environment often encourages youths to participate in alcohol use and engaging in heavy forms of drinking usually takes place in social gatherings [63]. These youths might be socially satisfied, as the cognitive discrepancy loneliness theory implies. The findings of this study might additionally reveal that among American and Finnish youths, drinking alcohol is not only a characteristic behavior of the socially active but behavior that could be considered as indulgent; an allowed form of gratification, related to having fun and enjoyment [34].

Gambling, even though often practiced alone, does not seem to be a lonely endeavor for the American or Finnish youths. Loneliness was associated with higher problem gambling in all of our country samples before adding offline and online belonging, and psychological distress, into the models. After accounting for these factors, the association remained significant only in South Korea. In Finland and the United States, other psychological factors, such as belongingness to friends or psychological distress, may be stronger predictors of problem behavior. For some youths, living alone might function as a noteworthy risk factor in both, youth compulsive internet use and problem gambling, as associations between these variables were found in 2 of the samples: the United States and South Korea. It is also possible that these 2 activities are related; due to the various online gambling opportunities, gambling activities add to the overall time spent online, thus contributing to compulsive internet use. Moreover, comorbidity is a common feature of addiction and it should be acknowledged that the addictive behaviors investigated in this study might co-occur among young individuals [64,65].

South Korean culture is described as more long-term oriented than those of the United States or Finland [34]. From this cultural perspective, it is possible that lonely South Korean youths are more distressed about how their perceived loneliness might disrupt their long-term goals, which manifests in behaviors that are counterintuitive for future well-being. In addition, loneliness may be a more serious issue in a collectivistic culture: those youths who feel loneliness may experience it much direr than their counterparts in less collectivistic countries.

### Limitations

Notwithstanding the strengths of the study, some limitations must be acknowledged. First, the study utilized a cross-sectional design that does not allow for concluding that the examined associations have a directional causal relationship. Second, our results show some differences across the 3 countries but cannot pinpoint what makes the differences. Future research should explore various psychological and sociocultural factors that may contribute to the differences. Finally, the problem of self-reported survey data on substance use or illicit behavior must be acknowledged as a research limitation, as these questions can be sensitive and lead to social desirability bias.

### Implications

Loneliness is an encompassing and harmful experience that can impact an individual on both physical and mental levels [27,28]. Although much research has been done on the effects of loneliness on adult populations, adolescents and emerging adults are also vulnerable populations to the harms of loneliness, as well as increasingly susceptible to destructive behaviors. Therefore, examining the relationship between these 2 variables is highly important. This study analyzed multicultural data on youths aged 15 to 25 years and found significant associations between perceived loneliness and addictive behaviors, thus adding to the existing literature. These results indicate that, across diverse cultures, youths who experience loneliness also report higher engagement in destructive behavior, especially that of compulsive internet use.

On the basis of these results, it is suggested that prevention methods for compulsive internet use be improved by educating youths about the harms of excessive online usage. An existing challenge is to reach young compulsive internet users. One promising direction of prevention is the development and building of internet-based, culturally informed behavioral intervention technologies that provide real-time support and personalized feedback, as well as assist with self-monitoring about the time elapsed online [66,67]. Furthermore, a need to develop youth prevention programs to lessen experienced loneliness is recognized. Youths should also be informed about how behaviors that may seem mundane and part of the everyday life can become excessive and disrupt healthy functioning. Focusing on strengthening youths’ social relationships and educating them about the harms of loneliness are additional main implications suggested by this research.

### Conclusions

Analyzing data from 3 countries, this research is able to add valuable and cross-culturally comparative insight into youth addictive behaviors. By applying social psychological theory on the phenomenon, this study extends the existing understanding on youth addiction. It can offer new information for policy makers and those working with young individuals on how to better address specific youth problem behaviors. Country and culture seem to have their share in influencing and shaping the underlining reasons, or ways, as to why the youth come to engage in certain behaviors. It is emphasized that youths coming from and living in different contexts might externalize their feelings of loneliness in different ways. Although the internet is used compulsively by youths in countries across the globe, South Korean youths also might externalize feelings of loneliness by engaging in excessive drinking and problem gambling. These youths might benefit from support and counseling that guides them toward other healthier activities.

This study expands youth addiction research beyond a national boundary. It offers additional insight into how perceived loneliness relates to 3 different types of youth addictive behaviors. Implications suggest improving prevention methods in compulsive internet use by educating youths about the harms of experienced loneliness and forms of excessive behaviors.

## Appendix

Multimedia Appendix 1 Complementary correlation and zero-inflated Poisson regression analyses.

Multimedia Appendix 2 Complementary logistic regression analysis.

## Abbreviations

AUDIT-C: Alcohol Use Disorders Identification Test-Consumption
CIUS: Compulsive Internet Use Scale
OLS: ordinary least squares
SOGS: South Oaks Gambling Screen
