# Supplementary material for: The Role of Perceived Loneliness in Youth Addictive Behaviors: Cross-National Survey Study
Source: JMIR Ment Health. 2020 Jan 2;7(1):e14035. doi: 10.2196/14035 (PMC6966551; doi:10.2196/14035)
Supplement: Multimedia Appendix 2 [file mental_v7i1e14035_app2.docx]

Multimedia Appendix 2.

Complementary logistic regression analyses.

Table 8. Logistic regression analyses on the effects of loneliness on excessive alcohol use (AUDIT-C), compulsive Internet use (CIUS), and problem gambling (SOGS) in three countries.

|  |  | UnitedStates |  |  | South Korea |  |  | Finland |  |
| --- | --- | --- | --- | --- | --- | --- | --- | --- | --- |
| Source | OR^b^ | SE | 95% CI^c^ | OR | SE | 95% CI | OR | SE | 95% CI |
| Excessive alcohol use | 1.05 | .04 | 0.97, 1.15 | 1.25^a^ | .06^a^ | 1.15, 1.37^a^ | .96 | .04 | 0.88, 1.04 |
| Compulsive internet use | 1.41^a^ | .06^a^ | 1.30, 1.52^a^ | 1.24^a^ | .05^a^ | 1.14, 1.35^a^ | 1.20^a^ | .05^a^ | 1.10, 1.31^a^ |
| Problem gambling | 1.03 | .10 | 0.86, 1.23 | 1.25 | .18 | 0.95, 1.65 | 1.10 | .11 | 0.90, 1.32 |

Note. Dependent variables are addictive behaviors and reported independent variable was loneliness. All models were run separately for each country and addiction type. Models are adjusted for age, gender, living alone, belonging to friends, belonging to online community, and psychological distress.

^a^Statistically significant results (*P*<.05).

^b^Odds ratio.

^c^ 95% confidence interval.
